# English as the Speech of the Future

**Published:** 1882-05

**Authors:** 


### ENGLISH AS THE SPEECH OF THE FUTURE.

The success of the English-speaking people as colonists and their superior prolificness are not the only reasons for thinking that the English tongue is destined to dominate the world. The flexibility and terseness of the English language has made it the language of international telegraphy, and from statistics just collected it appears to be the great newspaper language. In other words, it about equally divides the newspapers of the world with all other tongues combined.

The total number of newspapers and periodicals now published is given in H. P. Hubbard's forthcoming "Newspaper and Bank Directory of the World," as 34,376, with a circulation of about 116,000,000 copies, the annual aggregate circulation reaching in round numbers 10,600,000,000 copies. Europe leads with 19,557, and North America follows with 12,400, the two together making over nine-tenths of all the publications in existence. Asia has 775, South America 699, Australia 661, Africa 131. Of all these 16,500 are printed in the English language, 7,800 in German, 3,850 in French and over 1600 in Spanish. There are 5,020 daily newspapers, 18,274 tri-weeklies and weeklies, and 8,508 issued less frequently. It appears that while the annual aggregate circulation of publications in the United States is 1,600,000,000, that of Great Britain and Ireland is 2,260,000,000.

